# The development of ambivalent sexism: Proposals for an expanded model

**DOI:** 10.1111/bjdp.12521

**Published:** 2024-09-14

**Authors:** Campbell Leaper

**Affiliations:** ^1^ Department of Psychology University of California, Santa Cruz Santa Cruz California USA

**Keywords:** adolescence, gender attitudes, gender nonbinary, gender nonconformity, sexism, sexual identity, transgender

## Abstract

The United Nations' Goals for Sustainable Development highlight gender inequality as a pervasive problem around the world. Developmental psychologists can help us understand the development and consequences of sexism in people's lives. I highlight ambivalent sexism theory as a promising framework for this work; and I offer recommendations for expanding the theory. Ambivalent sexism theory distinguishes between hostile sexism and benevolent sexism as complementary processes perpetuating and maintaining men's dominance and heteronormativity in society. I summarize how these two forms of sexism emerge during childhood and adolescence; and I review the negative manifestations of hostile and benevolent sexism during adolescence and adulthood. Next, I chart several directions for expanding the ambivalent sexism model. These include addressing sexism directed towards gender‐nonconforming, sexual‐minoritized, and gender‐minoritized youth (in addition to sexism towards girls and women); taking into account the gender and sexual identities of both perpetrators and targets of sexism; considering a broader array of hostile and benevolent sexist practices than captured in existing measures; taking into account cultural variations and intersectionality in how ambivalent sexism is enacted; conducting more research on ambivalent sexism in childhood and adolescence and designing effective programs to reduce and to prevent ambivalent sexism beginning in childhood.

Gender equality is one of the United Nations' 17 Goals for Sustainable Development (United Nations, 2024). However, gender inequality remains a pervasive problem worldwide across regions, income levels and cultures (United Nations Development Programme, 2023, p. 3). Hence, understanding the causes and consequences of sexism remains a critical goal for scientists. Most research on sexism has focused on prejudice and discrimination targeted towards girls and women. In addition, sexism is commonly directed at persons with non‐heteronormative sexual identities (e.g. lesbian, gay, bisexual, queer), gender‐expansive identities (e.g. transgender, nonbinary, gender fluid), or gender‐nonconforming expressions (e.g. cisgender/heterosexual youth with gender‐nonconforming appearances, interests or behaviours). Furthermore, particular manifestations of sexism can vary across differing cultural and minoritized communities (Hayes & Swim, 2013; Leaper & Gutierrez, 2024).

In developmental psychology, theory and research on the causes and consequences of sexism have dramatically increased over the last two decades (e.g. Bigler & Liben, 2006; Brown et al., 2020; Brown & Bigler, 2005; de Lemus et al., 2015; Leaper, 2000; Leaper & Brown, 2018; Ramiro‐Sánchez et al., 2018; Robnett & Vierra, 2023). Families, peers, neighbourhoods, schools, media and other microsystems establish and maintain practices that perpetuate sexism in children's and adolescents' lives in multiple ways (Brown et al., 2020; Rogers et al., 2021). First, gender categories are regularly used to label and organize particular activities as ‘for girls’ or ‘for boys’ (Bigler & Liben, 2006; Leaper & Bigler, 2018). Second, children commonly segregate into same‐gender peer groups that reinforce gender conformity (Leaper, 2022). Third, cultural norms and practices reinforce expectations that boys and men exhibit dominance and hide weakness, known as *hegemonic masculinity* (Connell & Messerschmidt, 2005). Relatedly, aggressive behaviours are regularly tolerated and reinforced in boys (Farkas & Leaper, 2016b). Fifth, cultural expectations of heterosexuality, known as *hegemonic heteronormativity*, reinforce traditional heterosexual roles and stigmatize those who do not conform (Harvey et al., 2020). This is related to the pervasive sexualization, objectification and sexual harassment of girls and women (Brown et al., 2020; Daniels et al., 2020). Finally, gender biases in academic and other achievement contexts from childhood into adulthood contribute to women's underrepresentation in many high‐paying occupations, which perpetuates gender inequalities in income and occupational status (Cheryan et al., 2017; Kessels, 2023; Leaper & Brown, 2018).

There are multiple theories that are relevant to understanding particular facets of sexism and its development. In this paper, I will focus on ambivalent sexism theory (Glick & Fiske, 1996, 2012). The goal of the present paper is to propose an expanded model of this theory to study and conceptualize the development of sexism from childhood into adulthood. Whereas the original theory focused on sexism in the context of heterosexual relationships, I believe it can be readily extended to sexism directed at sexual‐ and gender‐minoritized youth and adults. Furthermore, I will suggest additional ways to expand the theory. Before offering my recommendations, I provide an overview of the ambivalent sexism theory and major research findings based on the theory.

## OVERVIEW OF AMBIVALENT SEXISM THEORY AND RESEARCH

In 1996, Glick and Fiske presented their *ambivalent sexism theory* that distinguishes between hostile sexism and benevolent sexism (see Bareket & Fiske, 2023; Connor et al., 2017; Hammond & Overall, 2017, for comprehensive reviews; also see Table 1 for an overview). Their model sought to reconcile the inherent ambivalence in heterosexual relations wherein women and men traditionally form intimate relationships predicated on gender‐based power and status differences favouring men. *Hostile sexism* comprises overtly negative attitudes and behaviours towards women – especially those who do not adhere to the traditional gender hierarchy. *Benevolent sexism* is based on patronizing attitudes and behaviours that are often seen as positive by men and women – yet reinforce women's subordinate status. To assess individuals' hostile and benevolent attitudes, Glick and Fiske (1996) formulated the ambivalent sexism inventory (ASI) based on samples of adults (see Table 1 for sample items). The ASI has since been used with adolescent samples (see de Lemus et al., 2015; Leaper & Gutierrez, 2024, for reviews).

**TABLE 1:** Expanding ambivalent sexism model: differentiating target of sexism and adding gender‐nonconforming persons.

Ambivalent sexism towards women^a^			
	Paternalism	Gender differentiation	Heteronormativity
**Hostile sexism towards women**	**Dominative paternalism**	**Competitive gender differentiation**	**Heterosexual hostility**
Description	Assertion of men's power and privilege	View that only men are suitable for high‐status positions	Controlling and demeaning views of women's sexuality
Sample Scale Item^a^	‘The world would be a better place if women supported men more and criticized them less’	‘A wife should not be significantly more successful in her career than her husband’	‘There are many women who get a kick out of teasing men by seeming sexually available and then refusing male advances’
**Benevolent sexism towards women**	**Protective paternalism**	**Complementary gender differentiation**	**Heterosexual intimacy**
Description	Chivalrous expectations that women need men's protection	Women/men have complementary traits but men's higher in power (e.g. assertive vs. nurturing)	Women complete men in heterosexual relationships; idealized view of women
Sample Scale Item^a^	‘Women should be cherished and protected by men’	‘Women, as compared to men, tend to have a more refined sense of cultural and good taste’	‘A man is not truly complete as a person unless he has the love of a woman’

Ambivalence towards men^b^			
	Paternalism	Gender differentiation	Heteronormativity
**Hostility towards men**	**Resentment of paternalism**	**Compensatory gender differentiation**	**Heterosexual hostility**
Description	Resenting men's greater power and privilege	Highlighting women's strengths relative to men	Resentment of men's aggressiveness
Sample Scale Item^b^	‘Men will always fight to have greater control in society than women’	‘Men would be lost in this world if women weren't there to guide them’	‘Men have no morals in what they will do to get sex’
**Benevolence towards men**	**Maternalism**	**Complementary gender differentiation**	**Heterosexual intimacy**
Description	View that men need nurturing and care	Admiration for men's higher status and abilities	View that fulfilment requires a romantic relationship with a man
Sample Scale Item^b^	‘Women ought to take care of their men at home, because men would fall apart if they had to fend for themselves’	‘Men are less likely [than women] to fall apart in emergencies’	‘Every woman needs a male partner who will cherish her’

Ambivalent sexism towards gender‐nonconforming persons^c^			
	Paternalism	Gender differentiation	Heteronormativity
**Hostile sexism towards gender‐nonconforming persons** ^c^	**Dominative paternalism**	**Competitive gender differentiation**	**Heterosexual hostility**
Description	Gender‐based bullying	View of nonconformity as inferior	Hostility towards gender‐nonconforming or sexual‐minoritized persons
**Benevolent sexism towards gender nonconforming persons** ^c^	**Protective paternalism/maternalism**	**Complementary gender differentiation**	**Heterosexual intimacy**
Description	Pity and efforts to change gender/sexual nonconformity	Benevolent stereotypes	Benevolent interactions with gender‐non‐onforming or sexual‐minoritized persons

^a^ Ambivalent sexism model (Glick & Fiske, 1996).

^b^ Ambivalence towards men model (Glick & Fiske, 1999).

^c^ Ambivalent sexism towards gender‐nonconforming persons is proposed in the current paper as an expansion of the ambivalent sexism model. Depending on the research question, the focus could be on gender‐nonconforming persons who are cisgender/heterosexual, gender‐minoritized persons, or sexual‐minoritized persons.

When conceptualizing and measuring hostile and benevolent forms of sexism in adult samples, Glick and Fiske (1996) proposed three facets that underlie each type of sexism (see Table 1): *paternalism* (viewing men as authority figures), *gender differentiation* (valuing gender‐based roles) and *heterosexuality* (i.e. holding heteronormative expectations). For hostile sexism, the corresponding types of each facet are *dominative* paternalism (affirmation of men's power), *competitive* gender differentiation (belief that inherent men's traits are superior for high‐status positions), and heterosexual *hostility* (controlling and demeaning views of women's sexuality). For benevolent sexism, the respective types of each facet are *protective* paternalism (chivalrous attitudes that women need protecting), *complementary* gender differentiation (beliefs that women and men have complementary traits that asymmetrically favour men for power and status [e.g. aggressive vs. nurturing]), and heterosexual *intimacy* (holding an idealized view that women complete men in heterosexual relationships [i.e. heteronormativity]). Thus, ambivalent sexist attitudes reflect an ideology that reconciles the paradox of women and men entering intimate heterosexual relationships that are predicated on men's dominance (Connor et al., 2017; Glick & Fiske, 1996).

Glick and Fiske's (1996) factor analysis of items in the ASI confirmed a three‐factor model for benevolent sexism; however, a single‐factor model best reflected hostile sexism (i.e. the three facets were not distinctive factors). Although some researchers have examined specific facets (particularly for benevolent sexism), most investigators have utilized the composite hostile sexism scale and the composite benevolent sexism scale in their analyses (Bareket & Fiske, 2023). Individuals' hostile and benevolent sexist attitudes were moderately correlated with one another across samples of predominantly heterosexual men and women in a variety of countries (Glick et al., 2000, 2004; Glick & Fiske, 2012); however, people were more likely to endorse benevolent sexism than hostile sexism (Connor et al., 2017). Also, a cross‐national comparison found that adults' endorsements of ambivalent sexism were less likely in more gender‐egalitarian countries (Glick et al., 2000).

Glick and Fiske (1999) elaborated on their ambivalent sexism theory when they proposed a model of women's *ambivalence towards men*. This model focused on the mixed attitudes that many women experience in response to men's sexism. To assess these attitudes, they designed the ambivalence towards men inventory (AMI) to distinguish between hostility towards men and benevolence towards men (see Table 1 for sample items). Hostility towards men includes *resentment of paternalism* (resenting men's greater power and status), *compensatory gender differentiation* (highlighting women's socioemotional strengths relative to men) and *heterosexual hostility* (resentment of men's sexual aggressiveness). Benevolence towards men comprises *maternalism* (the belief that men need nurturing and care), *complementary gender differentiation* (admiration for men's higher status and abilities) and *heterosexual intimacy* (the belief that fulfillment requires a romantic relationship with a man). Ambivalence towards men reflects the resentments and rationalizations that women can experience while negotiating traditional gender roles.

Relatively fewer studies have investigated women's ambivalence towards men compared to the numerous studies based on the original ASI. Later in my review, I suggest some possible directions for an integrative model that considers how hostile and benevolent sexism might affect attitudes depending on individuals' gender and sexual identities as well as the identities of the persons to whom their attitudes are directed. To establish the importance of understanding the development of ambivalent sexism in childhood and adolescence, I next review the multiple ways that ambivalent sexism may negatively affect adults.

### Correlates of hostile and benevolent sexist attitudes in adults

As documented in Bareket and Fiske's (2023) systematic literature review, both hostile and benevolent sexist attitudes are related to a variety of outcomes that maintain and perpetuate gender inequalities. To provide background on the impact of ambivalent sexism, I will summarize the major patterns that they identified. These studies were largely based on samples of adults who were cisgender and heterosexual. I will later note similar patterns observed among adolescents. I will also discuss the relevance of the ambivalent sexism model for gender‐ and sexual‐minoritized youth.

First, Bareket and Fiske (2023) reported ambivalent sexist attitudes were linked to *system‐justifying ideologies and endorsements of traditional gender* among both men and women. Hostile sexism was associated with the justification of male dominance and the affirmation of traditional norms of masculinity, and benevolent sexism was tied to the justification of traditional gender roles and the support of traditional norms of femininity. In addition, hostile and benevolent sexism predicted negative attitudes towards lesbian, gay, bisexual and transgender persons.

Second, ambivalent sexist attitudes were associated with holding *stereotypical perceptions of women and men*. Men's and women's hostile sexism predicted negative stereotypes about women (e.g. manipulative), negative evaluations towards nontraditional women and negative evaluations towards nontraditional men. Also, men's hostile sexism predicted their sexual objectification of women. Conversely, men's and women's benevolent sexism was correlated with positive stereotypes about women (e.g. warm) and favourable evaluations of traditional women. Women's benevolent sexism was additionally associated with their self‐objectification or positive views towards objectification.

Third, benevolent and hostile sexist attitudes predicted men's and women's *heterosexual relationships* in multiple ways. Benevolent sexist attitudes were associated with men's and women's preferences for traditional partners, traditional dating and family roles and relationship satisfaction when both partners' preferences aligned with traditional gender roles. However, women's endorsement of benevolent sexism may decline over time in relationships. Men's hostile sexist attitudes predicted their preference for traditional partners, men's endorsement of sexual double standards, men's traditional power‐related norms and men's and women's relationship dissatisfaction.

Fourth, ambivalent sexist attitudes were linked to *sexual harassment and violence against women*. Hostile sexism predicted men's likelihood of sexually harassing women or committing violence against women. Both hostile and benevolent sexism predicted both men's and women's tolerant attitudes towards sexual harassment and violence against women. In a recent meta‐analysis, the association between *benevolent* sexist attitudes and tolerant attitudes towards violence against women was stronger for women than men (Gutierrez & Leaper, 2024a) – underscoring the pernicious impact of benevolent sexism. That is, benevolent sexism may lead women as well as men to hold tolerant attitudes regarding sexual harassment and sexual violence.

Finally, ambivalent sexism predicted *gender inequalities in the workplace*. Both men's and women's hostile sexist attitudes were related to their direct bias and discrimination against women (e.g. devaluing women's competence). Benevolent sexist attitudes were related to paternalistic attempts to promote women as long as they conformed to traditional gender roles. Also, women's benevolent sexist attitudes appeared to undermine their aspirations and performance.

In summary, extensive research has established that hostile and benevolent sexist attitudes are related to multiple negative outcomes among adults. From a developmental perspective, we need to understand how these attitudes and behaviours emerge during childhood and adolescence. That is, through gender‐differentiated socialization practices and gender‐inequitable opportunities in society, children and adolescents begin to internalize ambivalent sexist attitudes and enact sexist behaviours. I review evidence for these trends next.

### Ambivalent sexism in childhood and adolescence

As reviewed above, studies of hostile and benevolent sexist attitudes have been primarily conducted with samples of adults. However, soon after the theory was advanced, its relevance for understanding gender development in children and adolescents was proposed (Glick & Hilt, 2000). Below, I provide an overview of how ambivalent sexism develops during childhood and adolescence. First, I suggest how the foundations of ambivalent sexism emerge in childhood. Second, I review how ambivalent sexism is increasingly enacted during adolescence. Finally, I briefly summarize some of the correlates of ambivalent sexist attitudes among adolescents.

#### Emergence of ambivalent sexism during childhood

To my knowledge, only two published studies have examined children's attitudes based on the ambivalent sexism model. In one study with 3‐ to 11‐year‐olds in the United States, researchers studied children's recognition of paternalistic norms whereby men are supposed to protect women (Gutierrez et al., 2020). Evidence suggested that paternalistic attitudes tended to increase with age. In a second study, researchers adapted the ambivalent sexism inventory for use with children between 5‐ and 11‐year‐olds in the United States (Hammond & Cimpian, 2021). These researchers' findings suggest that hostile and benevolent sexist attitudes may emerge during childhood.

Although there has been little work with children explicitly based on the ambivalent sexism model, existing research on gender development is consistent with the premise that ambivalent sexism is established during childhood. As I reviewed at the outset of my paper, cisgender girls and boys typically begin in early childhood to internalize traditional gender stereotypes and attitudes regarding activities, traits and roles (see Leaper, 2015; Martin et al., 2002). Examples include viewing boys as tough and girls as sweet, or associating men with high‐status occupations and women with caregiver roles. In addition, dominance goals are more common among boys than girls in their respective same‐gender peer groups (Leaper, 2015, 2022). In contrast, appearance‐related concerns emerge early among girls (Daniels et al., 2020). These self‐concepts and attitudes become the bases for complementary gender differentiation in benevolent sexist attitudes and for competitive gender differentiation in hostile sexism. As previously noted, some children appear to endorse paternalistic norms (Gutierrez et al., 2020; Hammond & Cimpian, 2021). Relatedly, researchers have observed children tend to view men as providers and women as caregivers (Fulcher & Coyle, 2011; Sinno & Killen, 2009).

#### Enacting ambivalent sexism during adolescence

Hostile and benevolent sexist *behaviours* become more pervasive during adolescence. Once heterosexual adolescents begin dating, benevolent sexism is apparent in the adoption of traditionally paternalistic dating scripts, such as the boy being the one to initiate and pay for the date (Paynter & Leaper, 2016). Hostile sexism in the forms of sexual objectification and sexual harassment tends to rise from early to late adolescence (Brown et al., 2020). Although these behaviours are directed towards boys and girls, they are more often aimed at girls. In addition, gender‐nonconforming youths are especially likely targets of harassment.

#### Endorsing ambivalent sexism during adolescence

Adolescents' endorsements of hostile and benevolent sexist *attitudes* have been observed across the world, including countries in Africa, Asia, South America, Europe and North America (see Leaper & Gutierrez, 2024). Notably, researchers in Spain have carried out much of this work (see de Lemus et al., 2015). However, compared to the numerous studies with adult samples, less research has investigated ambivalent sexist attitudes in adolescence (see Leaper & Gutierrez, 2024; Ramiro‐Sánchez et al., 2018, for reviews). Studies of adolescents (and adults) have been predominantly based on cisgender and heterosexual youth.

Links between adolescents' ambivalent sexist attitudes and behaviours are implicated across several studies. Perhaps the most investigated of these topics is the association between adolescents' ambivalent sexist attitudes and their acceptance or perpetration of sexual harassment and sexual violence (see Ramiro‐Sánchez et al., 2018). In addition, hostile and benevolent sexist attitudes predicted adolescents' negative attitudes or bullying behaviours towards sexual‐ or gender‐minoritized persons (e.g. Carrera‐Fernández et al., 2020), traditional attitudes regarding heterosexual dating and marriage (Brett et al., 2023), unrealistic romantic beliefs (e.g. Fernández et al., 2021), and sexual risk‐taking (Ramiro‐Sánchez et al., 2018). Furthermore, adolescent girls' benevolent sexist attitudes were correlated with restricted academic or career aspirations (e.g. Farkas & Leaper, 2016a; Sáinz & Gallego, 2022) and greater involvement in housework (e.g., Malonda et al., 2017).

Finally, researchers have begun to investigate potential socialization experiences related to variations in adolescents' endorsements of hostile and benevolent sexist attitudes. Their studies suggest that ambivalent sexism in adolescents is commonly reinforced via family messages (e.g. Gutierrez et al., 2022; Montañés et al., 2012), peer norms (e.g. Endendijk, 2024; Mastari et al., 2022), practices in schools (e.g. Cordón Gómez et al., 2019; Cortina et al., 2022) and representations in popular media (e.g. Brewington et al., 2022; Stermer & Burkley, 2015).

#### Summary

Ambivalent sexism theory was formulated to explain how intimate heterosexual relations occur in the context of gender inequalities in power and status. The theory explains how hostile sexism and benevolent sexism function as a complementary system for maintaining gender inequalities in heterosexual relationships and maintaining men's dominance in society. Both hostile sexism and benevolent sexism are tied to several adverse psychological outcomes in adults (see Bareket & Fiske, 2023) and adolescents (see Leaper & Gutierrez, 2024; Ramiro‐Sánchez et al., 2018). Developmental research suggests that the internalization of ambivalent sexism begins in childhood and becomes actively practised during adolescence. In the remaining part of my review, I suggest a few ways to build on the ambivalent sexism model to advance a more comprehensive model of sexism during childhood, adolescence, and adulthood.

## TOWARDS AN EXPANDED MODEL OF AMBIVALENT SEXISM

In the rest of my paper, I propose six directions towards an expanded model of ambivalent sexism for research with children, adolescents, and adults. When referencing current formulations and measures of ambivalent sexism, I will refer primarily to Glick and Fiske's (1996, 2012) studies with adults; however, I will additionally explain how my recommendations extend to studies of adolescents and possibly children.

### Recommendation 1: Take into account the gender and sexual identities of both the persons holding sexist attitudes and the persons who are the targets of sexism

The original Ambivalent Sexism Inventory (ASI) was designed for use with women and men to understand how and why sexist attitudes and related behaviours occur in intimate heterosexual relationships (Glick & Fiske, 1996). The inventory also has been used to examine sexism related to adolescents' heterosexual relationships (see Leaper & Gutierrez, 2024; Ramiro‐Sánchez et al., 2018). Because the ASI focused on ambivalent sexism towards women, Glick and Fiske (1999) later created the Ambivalence Towards Men Inventory (AMI) to consider heterosexual women's ambivalent attitudes about men's sexism. These modifications underscore that it is helpful to consider the identities of both persons holding sexist views and the identities of the targets of sexism (e.g. Lachance‐Grzela et al., 2021; Leaper et al., 2022). I propose further extending the ambivalent sexism model beyond those with cisgender/heterosexual identities. That is, an expanded theory would additionally incorporate those with gender‐minoritized, sexual‐minoritized, and other gender‐nonconforming identities. Furthermore, the model would take into account the gender and sexual identities of both the persons holding sexist attitudes (i.e. the actors or perpetrators) and the targets of sexism.

First, an expanded model would address the gender and sexual identities of *the targets of sexism*. Besides examining sexism towards cisgender/heterosexual girls and women, I propose an extended version of the model that addresses sexist attitudes directed towards three broad groups who do not conform to traditional heteronormativity: (1) sexual‐minoritized persons (e.g. gay, lesbian, bisexual, queer, etc.), (2) gender‐minoritized persons (e.g. transgender, nonbinary, gender‐fluid, etc.) and (3) cisgender/heterosexual persons whose gender expressions (e.g. appearances, interests, values, behaviours) do not conform to traditional norms. Ambivalent sexist attitudes measured with the ASI were correlated with negative attitudes towards gender‐minoritized, sexual‐minoritized, and other gender‐conforming persons in studies with adolescents (e.g. Carrera‐Fernández et al., 2021) and adults (e.g., Nagoshi et al., 2019). However, creating inventories that specifically address attitudes towards these groups is needed.

Hostile sexism underlies prejudice and discrimination against sexual‐minoritized, gender‐minoritized, and other gender‐nonconforming persons. Hostile prejudice and discrimination against members of these groups are forms of dominative paternalism (e.g. the assertion of heterosexual male dominance), competitive gender differentiation (e.g., devaluing gender nonconformity), and heterosexual hostility (e.g. homophobic teasing of gender nonconformity). Extensive research has documented how gender‐ and sexual‐minoritized children, adolescents, and adults are commonly targeted for gender‐based harassment and bullying (see Brown et al., 2020; Leaper & Gutierrez, 2024; Mitchell et al., 2014).

In addition, benevolent sexism in the forms of complementary gender differentiation and protective paternalism are seen in many people's attitudes towards sexual‐ or gender‐minoritized persons (Bochicchio et al., 2024; Dixon & Langdridge, 2022; Massey, 2010) or other gender‐nonconforming individuals (Adams et al., 2016). Complementary gender differentiation includes holding seemingly positive yet patronizing views towards group members. An example might be stereotyping gay men as ‘stylish’ or ‘good listeners’ (e.g. Morrison & Bearden, 2007). Also, stereotypical yet seemingly positive depictions of LGBTQ+ persons are common in popular media (McInroy & Craig, 2017). Protective paternalism is conveyed via expressions of pity for sexual‐ or gender‐minoritized persons (Vaughn et al., 2017). A related question for future research is to consider if and how manifestations of hostile or benevolent sexism towards sexual‐minoritized youth or adults may differ depending on their respective gender identities (e.g. gay boys vs. lesbian girls).

Second, a fuller model of ambivalent sexism would take into account the gender and sexual identities of *the persons holding sexist attitudes (i.e. the actors or perpetrators)*. A few studies have evaluated the endorsements of hostile and ambivalent sexism in samples of gender‐minoritized and sexual‐minoritized adults (e.g. Cowie et al., 2019; Schiralli et al., 2022; Zheng et al., 2017; but also see Cross et al., 2021, regarding potential measurement equivalence issues). Whereas some members of these groups endorsed ambivalent sexist attitudes, they often were less likely to do so than heterosexual or cisgender persons. To my knowledge, no prior studies of children or adolescents have examined ambivalent sexist attitudes in samples of gender‐minoritized, sexual‐minoritized, or other gender‐nonconforming persons. Adolescence is typically a period when individuals are exploring their gender and sexual identities. Thus, for these youths, reconciling their gender‐nonconforming gender or sexual identities with sexist attitudes in the broader culture often poses challenges (e.g. deMayo et al., 2022; Robertson, 2014). As suggested in studies of adults, some gender‐ and sexual‐minoritized children and adolescents may internalize sexist attitudes.

In summary, I recommend revising the ambivalent sexism theoretical model to consider sexism directed towards gender‐minoritized, sexual‐minoritized, and other gender‐nonconforming persons. In doing so, researchers would additionally take into account the identities of both the persons holding sexist gender attitudes and the persons who are the targets of these attitudes. This expanded model could be useful in studying sexism in childhood and adolescence as well as adulthood. An abridged outline for my proposed model is presented in Table 1. In the table, I focus on the targets of sexism (and it does not include the perpetrators). In addition, I list gender‐conforming persons as a single group that includes gender‐minoritized, sexual‐minoritized, and other gender‐nonconforming persons. The more specific groups would be identified depending on the research questions of a given study.

### Recommendation 2: Elaborate on each of the three facets of ambivalent sexism

In the ambivalent sexism model, hostile sexism and benevolent sexism each comprise three facets: gender differentiation (complementary or competitive), paternalism (dominative or protective), and heteronormativity (i.e. heterosexual intimacy or hostility). As I explained earlier, Glick and Fiske (1999) found that the three‐factor structure worked for benevolent sexism in the ASI; but only a one‐factor structure was supported for hostile sexism. That does not necessarily exclude finding support for a three‐factor structure of hostile sexism with a different set of inventory items. For a fuller theoretical understanding of benevolent and hostile sexism, I encourage investigators to consider each of the three facets more deeply. In this way, researchers could examine variations in ways that facets of hostile and benevolent sexism are endorsed and practised across individuals – and, importantly, across cultural groups.

Research on traditional gender ideologies could help inform my recommendation to elaborate on the facets of ambivalent sexism. Traditional gender ideologies refer to people's beliefs and attitudes regarding masculinity and femininity that underlie their identities and guide their views and actions towards others. Several measures have been devised to identify and assess multiple facets of these ideologies (see Moradi & Parent, 2013; Thompson & Bennett, 2015). Many of these scales have been used to study adolescents (e.g. Levant et al., 2008; Oransky & Fisher, 2009; Tolman et al., 2006). Although different rubrics of traditional masculinity and traditional femininity have been advanced, they share some recurring themes (see Moradi & Parent, 2013). Traditional masculine ideology stresses dominance and status, self‐reliance and toughness, emotional restriction (of vulnerable feelings), the sexual objectification of women, anti‐femininity, heteronormativity and heterosexism. Traditional feminine ideology emphasizes caregiving and socio‐emotional roles, self‐deference and dependency, heteronormativity, appearance concerns, modesty, and sexual fidelity.

Many of the components in these models overlap conceptually and empirically with facets of hostile and benevolent sexism (e.g. Krivoshchekov et al., 2023). These include ideologies related to men's paternalism (e.g. dominance, status, emotional control), men's gender differentiation (aggression, toughness, self‐reliance, risk‐taking, winning), women's gender differentiation (emotionality, dependency, modesty/virtue, caretaking), men's heteronormativity (e.g. sexual entitlement, the importance of sex, sexual objectification, anti‐femininity) and women's heteronormativity (sexual fidelity, self‐objectification, orientation towards romantic relationships). Prior studies indicate that these beliefs are prevalent among adolescents (see Leaper & Gutierrez, 2024; Ramiro‐Sánchez et al., 2018) and likely emerge during childhood (e.g. Benenson et al., 2019; Fulcher & Coyle, 2011). Related works on sexual double standards (e.g. Endendijk et al., 2022) and traditional heterosexual dating and marital scripts (e.g. Brett et al., 2023; Paynter & Leaper, 2016) highlight themes related to complementary gender differentiation (e.g. girl or woman as chaste), protective paternalism (e.g. boy or man initiates and pays for date), and heteronormativity.

In addition to the gender ideology measures, I recommend incorporating an assessment of gender‐essentialist thinking in an expanded ambivalent sexism model (e.g. Fine et al., 2024; Lee et al., 2020). *Essentialism* refers to beliefs that particular characteristics are inherent and stable essences of group members. Regarding gender attitudes, examples include beliefs that boys are naturally aggressive (‘boys will be boys’) or girls are naturally nurturing (‘sugar and sweet and everything nice’). Gender‐essentialist thinking is prevalent among children (e.g. Taylor et al., 2009); yet, it is also seen among many adolescents and adults; and essentialist thinking can lead to sexist prejudices (e.g. Atwood et al., 2024; Bigler & Liben, 2006; Fine et al., 2024; Meyer & Gelman, 2016). Essentialist thinking is also implied in the complementary gender differentiation facet in the ambivalent sexism model (e.g. ‘Many women have a quality of purity that few men possess’ [Glick & Fiske, 1996, p. 496], ‘Men are more willing to take risks than women’ [Glick & Fiske, 1999, p. 527]). Although implied, essentialism is not explicitly assessed in the ASI or AMI. A recently proposed measure of gender‐essentialist thinking (Lee et al., 2020) could be used in research seeking to advance an expanded ambivalent sexism model.

In summary, I propose expanding the ambivalent sexism model to consider a broader range in the ways that paternalism, gender differentiation and heteronormativity are manifested in hostile and benevolent sexism. In this manner, we can enrich ambivalent sexism theory and research. By now, the reader may be wondering about the practicality of my proposal. If the ambivalent sexism inventory was extended to reflect my suggestions, it would imply a very lengthy inventory. Instead, I recommend studying expansions of one or a few facets at a time to advance theory development. Over time, a comprehensive ambivalent sexism inventory might follow. As I discuss next, researchers may additionally consider using alternative versions of an ambivalent sexism inventory in different sociocultural contexts.

### Recommendation 3: Consider cultural variations and intersectionalities in how hostile and benevolent sexism are enacted

Across the world, countries vary in their degrees of overall gender equality (United Nations Development Programme, 2023; World Economic Forum, 2024). To compare nations, the metrics used in the global gender gap index are economic participation and opportunity, educational attainment, political empowerment and health and survival rates (World Economic Forum, 2024). Nordic countries such as Iceland, Denmark and Sweden have generally ranked atop these rankings in gender equality. Several studies have documented ways that average gender differences in children's or adults' abilities, attitudes and behaviours vary depending on a country's ranking on indices of gender equality (see Herlitz et al., 2024). Notably, however, no country in the world has attained full gender equality. To wit, ambivalent sexist attitudes have been documented among adolescents in Sweden (Zakrisson et al., 2012).

Besides considering cultural variations, little attention has been paid to regional variations in gender norms and practices on the development of sexist attitudes. For example, there are variations across regions of the United States in attitudes and laws regarding the rights of women and gender‐diverse persons, which may differentially affect children's developing gender attitudes and behaviours. Similarly, the United Nations Gender Development Index has documented many within‐country variations in gender inequality across the world (Smits, 2020).

In addition to within‐country variations, the meaning and manifestations of hostile and benevolent sexism may vary to some extent across groups based on their cultural backgrounds, race, class and other social standings within a society (see Leaper & Gutierrez, 2024; Moradi & Parent, 2013). For example, researchers have noted that emphases on family, community closeness and respect for authority are embedded in the gender attitudes traditionally associated with families in the United States with backgrounds from Mexican and other Latiné cultures (e.g. Arciniega et al., 2008; Castillo et al., 2010; Gutierrez & Leaper, 2022) as well as Chinese and other East Asian cultures (e.g. Tang et al., 2010). Accordingly, some investigators have devised measures of gender attitudes designed for particular cultural groups.

To illustrate, I will describe the Traditional Machismo and Caballerismo Scale (Arciniega et al., 2008) and the Marianismo Beliefs Scale (Castillo et al., 2010). These instruments were designed for Latiné groups in the United States to assess traditional masculinity and femininity, respectively. The norms reflected in these culture‐based measures of gender attitudes are compatible with the ambivalent sexism model (e.g. Herrera Hernandez & Oswald, 2024). For example, attitudes assessed in the Machismo and Marianismo scales reflect paternalism (e.g. ‘In a family, a father's wish is law’), complementary gender differentiation (e.g. ‘It would be shameful for a man to cry in front of his children’ or ‘[Women should] be forgiving in all aspects’ for women) and heteronormativity (e.g. ‘[A woman should] remain a virgin until marriage’). However, a recent factor analysis indicated some subscales of the Machismo and Marianismo scales were distinct from the subscales in the ASI (Gutierrez & Leaper, 2024b). This finding lends support to my proposal to expand how we conceptualize and assess facets of ambivalent sexism.

Considering variations in the ways that ambivalent sexism may be manifested in particular cultural communities is compatible with intersectional approaches that consider the interconnections of multiple minoritized social identities, such as gender, sexuality, race, ethnicity and class (e.g. Ghavami et al., 2016). That is, forms of hostile and benevolent sexism may intersect with other identity‐based prejudices (e.g. McMahon & Kahn, 2016). To help inform these efforts and advance a more intersectional model of ambivalent sexism, researchers may additionally seek to integrate work on ambivalent racism (e.g. Katz & Hass, 1988) and ambivalent classism (Sainz, 2023) into an expanded ambivalent sexism theory.

In summary, ambivalent sexism has been documented across different cultural, ethnic and racial groups. Rather than seeking to identify universal gender ideologies, an alternative approach has been to consider particular ways that traditional femininity and masculinity ideologies are expressed in particular cultural communities. In addition, more consideration of intersectional perspectives is needed whereby forms of hostile and benevolent sexism may interact with other identity‐based prejudices such as racism and classism. Accordingly, I recommend incorporating cultural and intersectional approaches into an expanded ambivalent sexism theory.

### Recommendation 4: Study the development and correlates of ambivalent sexist attitudes in childhood and adolescence

I have argued in this paper that the distinction between hostile sexism and benevolent sexism with their corresponding facets can serve as a useful framework for conceptualizing how and why sexism develops during childhood and adolescence. Very little work has examined the development and impact of ambivalent sexism in childhood. However, research on ambivalent sexism in adolescence has grown. As I summarized earlier, youths' hostile and benevolent sexist attitudes have been linked to bullying sexual‐ and gender‐minoritized youth, sexual harassment, sexual objectification, intimate partner violence and restricted academic or career aspirations (see Leaper & Gutierrez, 2024; Ramiro‐Sánchez et al., 2018, for reviews).

From a developmental ecological‐systems perspective of gender development (e.g. Leaper, 2000), it would be helpful to learn more about the macro‐, meso‐ and micro‐systems that foster the internalization of ambivalent sexist attitudes from childhood into adolescence. Earlier, I cited studies pointing to the potential influences of families, peers, schools and media in the formation of ambivalent sexist attitudes. In conducting this research, I encourage researchers to consider the ambivalent sexist attitudes of potential socializing agents (i.e. perpetrators of sexism) such as parents, teachers and peers as well as the children and adolescents who are targets of socialization (e.g. Overall et al., 2023). Also, some topics that could use more attention include the impact of gender‐segregated peer groups on the development of ambivalent sexism (see Leaper, 2022) as well as the relations of ambivalent sexism to gender‐related variations in academic aspirations (see Leaper & Brown, 2018) and body image (see Daniels et al., 2020).

### Recommendation 5: Design new measures to assess ambivalent sexism

If researchers follow my advice to investigate a wider range of ambivalent sexist attitudes and behaviours, they will likely be designing new measures (to complement the ASI and AMI). I have four recommendations for those pursuing this work.

First, when trying to identify new facets of ambivalent sexism, researchers may want to start with qualitative methods, such as interviews and other narrative approaches, to explore how individuals construct and experience gender and sexism in their own lived experience (see Yoshikawa et al., 2013). This approach can reveal themes and issues that researchers may not have anticipated, which in turn can inform later quantitative research (e.g. survey construction).

Second, in designing new scale items, I advise using simple wording that avoids colloquial expressions. This will help make the scale accessible for younger samples and for individuals from a variety of cultural (and language) backgrounds. For example, one item in the ASI states ‘A good woman should be set on a pedestal by her man’ (Glick & Fiske, 1996, p. 512). The colloquial phrase ‘being put on a pedestal’ is likely understood by most readers, but it may be unfamiliar and unclear to some.

Third, items assessing people's gender attitudes should explicitly test whether individuals differ in the endorsements of behaviours based on people's gender. Gender attitude inventories such as the ASI typically ask participants to rate their agreement to statements regarding traditionally prescribed or proscribed traits, activities, or roles based on a person's gender (see Moradi & Parent, 2013). For example, one item reflecting protective paternalism in the ASI states, ‘Women should be cherished and protected by men’ (Glick & Fiske, 1996, p. 512). Endorsement of protective paternalism is inferred if the person agrees to the statement. However, it is possible that the respondent might similarly agree with the obverse statement that ‘Men should be cherished and protected by women’. If so, asymmetries in gender roles and protective paternalism would *not* be indicated. Therefore, I recommend utilizing wording formats that more explicitly evaluate whether individuals endorse particular behaviours differently based on a person's gender (see Paynter & Leaper, 2016, for further discussion and an example of a scale using an alternative method). To be clear, I am *not* suggesting that prior uses of the ASI are invalid. Extensive research has established its predictive validity (Bareket & Fiske, 2023). However, my suggested modifications could potentially mitigate occasional measurement error.

Finally, to investigate ambivalent sexist attitudes in children, researchers will need to devise developmentally appropriate methods. Although only two known studies have looked at ambivalent sexist attitudes in children (Gutierrez et al., 2020; Hammond & Cimpian, 2021), there has been extensive research on children's gender stereotyping and attitudes (e.g. Liben & Bigler, 2002). To measure children's gender attitudes, researchers have typically asked children whether particular gender‐stereotypical or counter‐stereotypical behaviours should be performed by a girl (or a woman), a boy (or a man), or either a girl or a boy (or either a woman or a man). In some instances, a vignette or a visual representation might be used to describe a behaviour. Using this approach, Gutierrez et al. (2020) found evidence of benevolent sexist attitudes in 3‐ to 11‐year‐old children. Another strategy is to read statements describing stereotypical or counter‐stereotypical depictions of gender and ask children to evaluate them (e.g. as ‘right’ or ‘wrong’). Using this method, Hammond and Cimpian (2021) modified some of the items from the ASI and found evidence of hostile and benevolent sexist attitudes in children 5–11 years of age.

In sum, I have offered a few methodological recommendations for future research on ambivalent sexism. I explained how qualitative methods could help inform the advancement of an expanded ambivalent sexism model. I also offered suggestions for wording items in new scales. Finally, I noted that research on ambivalent sexism in childhood will require utilizing developmentally appropriate methods.

### Recommendation 6: Design and evaluate programs to reduce and prevent ambivalent sexism

To promote gender equality, it is necessary to reduce structural sexism in society as well as foster gender‐egalitarian attitudes in people. As with most interventions designed to change people's attitudes and behaviours, it is usually better to start earlier rather than later in life. That is, it is more effective to prevent the formation of sexist attitudes and behaviours than to undo them. In this regard, some promising strategies for reducing gender‐based prejudice and discrimination have been developed for parents (e.g. Hilliard et al., 2021) and schools (e.g. Bigler & Pahlke, 2019; Brown & Salomon, 2019; Spinner et al., 2021). Most of these prior interventions focused on mitigating hostile forms of sexism (e.g. sexual harassment, demeaning views of girls or gender‐nonconforming youths). Many people who disavow hostile sexism, however, do not recognize benevolent sexism as problematic. Hence, it may be especially helpful to explain what it is and why it is a problem early in life. Consistent with this point, Bareket and Fiske's (2023) recent review noted that experimental interventions were generally more successful in reducing hostile sexism than benevolent sexism. To better reduce benevolent sexism, the authors highlighted programs that explicitly addressed the subtle, insidious and pervasive nature of benevolent sexism.

The intervention studies in Bareket and Fiske's review were conducted with adult samples. A few pilot programs with adolescents have been aimed at reducing their hostile and benevolent sexist attitudes and the likelihood of sexual violence (e.g. Carrascosa et al., 2019; Navarro‐Pérez et al., 2020). These programs involved increasing adolescents' awareness of hostile and benevolent sexism or having them systematically monitor their behaviours. Looking ahead, there is a clear need to continue designing and evaluating developmentally informed programs to reduce ambivalent sexist attitudes and behaviours in children and adolescents. Moreover, considering an expanded model of ambivalent sexism can guide these efforts.

## SUMMARY AND CONCLUSIONS

One of the major advances in the study of sexism in recent decades has been Glick and Fiske's (1996, 2012) formulation of ambivalent sexism theory and the design of their inventories. Whereas the theory was initially applied to studying sexism in adults, it subsequently guided numerous studies on sexism in adolescence. In research conducted across the world, measures of hostile and benevolent sexism have differentially predicted several negative outcomes in adolescents (see de Lemus et al., 2015; Leaper & Gutierrez, 2024; Ramiro‐Sánchez et al., 2018) and adults (see Bareket & Fiske, 2023; Connor et al., 2017). Nonetheless, it has been nearly 30 years since the theory and the inventories were first introduced. I offered a few recommendations to expand the theory and to chart new directions for research.

First, I advised taking into account the gender and sexual identities of both persons holding sexist attitudes and the persons who are targets of sexism in the ambivalent sexism model. Second, I noted potential areas for elaborating on ways that paternalism, gender differentiation and heteronormativity are manifested and assessed in the ASI. Third, I proposed considering cultural variations and intersectionalities in how hostile and benevolent sexism are enacted and reflected in the model. Fourth, I encouraged more research into the development and correlates of ambivalent sexism beginning in childhood. Fifth, I offered a few methodological suggestions for designing new scales to assess ambivalent sexism. Finally, I implored researchers to design programs to reduce ambivalent sexism in childhood and adolescence.

In light of the backlash to advances in gender equality in recent years (e.g. see Anderson, 2021), research on the development and consequences of sexism remains essential. The United Nations has identified attaining gender equality as one of its Goals for Sustainable Development (United Nations, 2024). Evidence suggests that attaining greater gender equality generally benefits all members of societies: Standards of living and happiness are generally higher in countries with greater gender equality (Chen et al., 2023; World Economic Forum, 2024).

## AUTHOR CONTRIBUTIONS

**Campbell Leaper:** Conceptualization; writing – original draft; writing – review and editing; investigation.

## CONFLICT OF INTEREST STATEMENT

The author declares there are no conflicts of interest.

## Data Availability

Not applicable.
